# Supplementary material for: Let-7b-5p sensitizes breast cancer cells to doxorubicin through Aurora Kinase B
Source: PLoS One. 2025 Jan 9;20(1):e0307420. doi: 10.1371/journal.pone.0307420 (PMC11717257; doi:10.1371/journal.pone.0307420)
Supplement: Attachment — Submitted filename: plos one response to reviewers_ZK.docx [file pone.0307420.s001.docx]

Dear Editor,

We thank the reviewers for their constructive and thoughtful comments and suggestions on our manuscript. We have revised the manuscript to address each of the comments. Below are our point-to-point responses to the reviewers’ comments.

The modifications in the manuscript are highlighted within the “Manuscript revised 07.10.2024_ZK.docx” document. We also provided an unmarked version of the revised manuscript “Manuscript 07.10.2024_Unmarked_ZK.docx” upon your request.

Sincerely,

Dr Zeyneb Kurt,

Lecturer in Data Science,

The University of Sheffield, The Information School

Additional Editor Comments:

**The reviewers provided very detailed suggestions for revisions. Many of them coincide with my views. The more important ones are as follows:**

**1- First, the manuscript relies to some extent on in silicon lab methods to advance, and it should consider adding in vivo experimental data to improve the limitations of this part of the method (or at least, explain it).**

**Response:**

The limitations of the study regarding bioinformatics data and the lack of in vivo experiments have been added to the discussion section of the study as follows:

“GEO2R is a web-based tool that is user-friendly and enables users to analyze various data sets from a GEO series in order to identify genes, miRNAs, circRNAs, and other molecules that are differentially expressed. Similarly, miRNet [1] is a web-based platform that is user-friendly and has been specifically designed to assist in the elucidation of miRNA effects by integrating users' data with existing knowledge through network-based visual analytics. Nevertheless, it is important to keep in mind that the results obtained using GEO2R, miRNET, and other in silico tools in the study may contain data that could restrict the study. For instance, in the GEO2R analysis, some miRNAs, excluded based on logFC and p-value, may have significant relevance in elucidating the link between BC and doxorubicin resistance. In our work, findings based on miRTarbase and Tarbase tools derived from the miRNET tool used to identify the possible target genes of the chosen miRNA (let-7b-5p). For the prediction of miRNA-Gene interactions, other target genes might be proposed for let-7b-5p in tools other than miRTarbase and Tarbase (e.g. miRDB). Thus, it is important to note that, in addition to the miRNAs and genes identified through various GEO datasets and in silico tools, certain significant miRNAs and genes may have been excluded due to the limitations of the bioinformatics methods employed. The investigation provided significant data regarding the relationship between BC Dox resistance/let-7b-5p/AURKB; however, the lack of in vivo experiments represents a different limitation of the study.”

**2- The manuscript should consider discussing genes related to the AURKB gene. Or better yet, the relationship between this part of the genes and doxorubicin resistance.**

**Response:**

We would like to thank the editor for this valuable comment. The relationship between AURKB-related genes and other drug resistance, including doxorubicin, was discussed and added to the discussion section as follows.

“In current study a comparison of the possible target genes of let-7b-5p in the miRNet tool between TCGA-BC data, GSE100925, and GSE229571 BC GEO datasets revealed 14 genes in common. The STRING analysis revealed a strong link between these 14 genes. Furthermore, a significant association was identified between AURKB and the other 13 genes. These genes have been shown to possess potential oncogenic roles in several malignancies, including BC. For instance, Huang et al. showed that CKS2 overexpression is a poor prognostic marker and promotes cell proliferation and invasion in BC [2]. Overexpression of CKS2 has been shown to enhance the effectiveness of chemotherapy by overriding DNA damage regulators [3]. It has been determined that upregulation of NUSAP1 expression stimulates cell proliferation in invasive BC cells via CDK1 and DLGAP5 and is related to Epirubicin resistance [4]. CCNA2 serves as a possible indicator for immunotherapy in BC and acts as a prognostic indicator for ER+ BC and tamoxifen resistance [5, 6]. RRM2 is another prognostic indicator in BC and is associated with worse survival outcomes and tamoxifen resistance [7]. RRM2 silencing with siRNA has been shown to significantly inhibit pancreatic tumor proliferation, either alone or with Dox [8]. CDCA8 is another prominent gene that may be related to AURKB. It is a key modulator of estrogen-induced cell proliferation in BC cells [9]. The AURKB/CDCA8 pathway is potentially pivotal in cancer pathogenesis. Hayama et al. showed that the phosphorylation and activation of CDCA8 by AURKB contribute to human lung carcinogenesis [10]. PLK1, whose increased expression is a major problem in BC, has been reported to mediate resistance to Palbociclib in HR+/HER2- metastatic BC [11]. According to studies, BIRC5 (surviving), an inhibitor of apoptosis (IAP) family member was substantially expressed in BC patients and, it also promotes resistance to chemotherapy, anti-HER2 treatment, and radiotherapy [12]. Mitotic kinases PLK1 and AURKB control BC cell proliferation by phosphorylating BIRC5 [13]. The literature that was assembled here suggests that the molecular linkage of genes connected to AURKB should be examined in more depth. This would allow for a better understanding of the processes that cause drug resistance in BC, as well as the development of novel treatments. Based on these findings, it has been suggested that the let-7b-5p/AURKB axis may affect cancer processes by influencing several key genes.”

**3- The manuscript should consider answering the relationship between AURKB and breast cancer subtypes or other tumors.**

**Response:**

We would like to thank the editor for his valuable comment. The relationship between AURKB and breast cancer/other tumors was discussed and added to the discussion section as follows:

The AURKB gene is overexpressed in many cancer types including BC [14-16]. Pellizzari et al reported that inhibition of the AURKB pathway may be a promising strategy for the treatment and radiosensitization of Triple Negative Breast Cancer (TNBC) [17]. It has been demonstrated that AURKB stimulates epithelial-mesenchymal transition by stabilizing Snail1, resulting in basal-like breast cancer metastases [18]. AURKB is also closely associated with drug resistance. For instance, Liu et al., revealed that AURKB is related to paclitaxel resistance in BC cells through the PRKCE/RAB27B axis, suggesting a possible intervention target to reverse tumor drug resistance [19]. A different investigation has demonstrated that AURKB facilitates the development of tumors and carboplatin resistance via controlling the ERK pathway in neuroblastoma cells [20]. Some studies have also shown that miRNAs can specifically target AURKB and are associated with drug resistance in cancers. For example, miR-486-5p has been shown to regulate DNA damage inhibition and cisplatin resistance in lung adenocarcinoma via AURKB [21]. The results of these studies indicate that AURKB may be responsible for drug resistance in certain malignancies through various molecular mechanisms, such as miRNAs. The provided research supports that let-7b-5p may be linked to doxorubicin resistance in BC through AURKB.

**REVIEWERS’ COMMENTS**

Reviewer #1: The manuscript titled "Let-7b-5p sensitizes breast cancer cells to doxorubicin through Aurora Kinase B" presents an interesting exploration of the relationship between miRNA let-7b-5p, Aurora Kinase B (AURKB), and doxorubicin resistance in breast cancer. While the study touches on a crucial aspect of breast cancer therapy and drug resistance, several significant concerns need to be addressed to strengthen the overall quality and impact of the research.

o **The study relies heavily on bioinformatics analyses, which is a valid approach. However, the validation of in silico findings should be more robust, especially considering the clinical implications of the results. The current data from quantitative PCR and cell line studies, while promising, seem insufficient to conclusively establish the role of let-7b-5p/AURKB in doxorubicin resistance. Additional experiments, such as in vivo models or patient-derived xenografts, would provide stronger evidence.**

**Response:**

We would like to thank the reviewer for the fair criticism. If our study findings could be validated using "in vivo models or patient-derived xenografts," that would be excellent. Unfortunately, the project funding does not allow us to integrate these methods within the study. Following the reviewer's recommendation, this limitation has been included in the part on the discussion.

“The investigation provided significant data regarding the relationship between BC Dox resistance/let-7b-5p/AURKB; however, the lack of in vivo experiments represents a different limitation of the study.”

o **The Discussion could benefit from a more focused discussion that directly relates previous studies to the findings of this research. For instance, while other studies have linked let-7b-5p to drug resistance in different cancers, the manuscript should clearly distinguish how this study’s findings on AURKB add to or differ from the existing knowledge.**

**Response:**

We express our gratitude to the reviewer for this significant reminder. New literature examples have been added to the discussion section, specifically highlighting the relationship between AURKB and drug resistance.

“The AURKB gene is overexpressed in many cancer types including BC [14-16]. Pellizzari et al reported that inhibition of the AURKB pathway may be a promising strategy for the treatment and radiosensitization of Triple Negative Breast Cancer (TNBC) [17]. It has been demonstrated that AURKB stimulates epithelial-mesenchymal transition by stabilizing Snail1, resulting in basal-like breast cancer metastases [18]. AURKB is also closely associated with drug resistance. For instance, Liu et al., revealed that AURKB is related to paclitaxel resistance in BC cells through the PRKCE/RAB27B axis, suggesting a possible intervention target to reverse tumor drug resistance [19]. A different investigation has demonstrated that AURKB facilitates the development of tumors and carboplatin resistance via controlling the ERK pathway in neuroblastoma cells [20]. Some studies have also shown that miRNAs can specifically target AURKB and are associated with drug resistance in cancers. For example, miR-486-5p has been shown to regulate DNA damage inhibition and cisplatin resistance in lung adenocarcinoma via AURKB [21]. The results of these studies indicate that AURKB may be responsible for drug resistance in certain malignancies through various molecular mechanisms, such as miRNAs. The provided research supports that let-7b-5p may be linked to doxorubicin resistance in BC through AURKB.”

**o The mechanistic pathway connecting let-7b-5p and AURKB to doxorubicin resistance is not thoroughly explored. The manuscript would be significantly strengthened by elucidating how this miRNA-target interaction specifically influences cellular pathways leading to drug resistance. Details on the downstream effects of AURKB inhibition by let-7b-5p, such as alterations in apoptosis or cell cycle regulation, should be provided.**

**Response:**

We appreciate the reviewer's valuable suggestion. In the present study, as detailed in the materials and methods section, to elucidate the relationship between let-7b-5p and AURKB, different GEO Data sets were utilized to identify differentially expressed microRNAs and genes. The literature was thoroughly reviewed to identify more robust miRNAs and target genes. Subsequently, the expression levels of let-7b-5p and AURKB were examined in 72 breast cancer patient samples using qRT-PCR. Then, the functional effects of the let-7b-5p/AURKB axis in doxorubicin-resistant and parental cells were investigated (Proliferation, migration, and apoptosis methods were applied, but the cell cycle method could not be included in the study). The study could definitely have been enhanced and expanded through the utilization of various methodologies. Revealing the effect of this process on the downstream pathway of AURKB, as suggested by the reviewer, is a valuable approach. This valuable suggestion from the reviewer has been assessed as a new project proposal by our working group.

**o The results would benefit from a more detailed and systematic presentation of the data. For example, the relationship between let-7b-5p and AURKB in both parental and doxorubicin-resistant cell lines should be explicitly compared and contrasted. Additionally, the inclusion of statistical analyses for all experiments would help clarify the significance of the findings.**

**Response:**

We would like to thank the reviewer for this suggestion. In the current study, as detailed in the material method, mimic let-7b-5p transfection was performed and AURKB inhibitor was used in parental and doxorubicin resistant cells. Bioinformatic analyses and subsequent breast cancer patient samples revealed that let-7b-5p was downregulated and AURKB was overexpressed. In the literature review, it was determined that let-7b-5p is a tumor suppressor in cancers and the AURKB gene exhibits oncogenic properties. Moreover, the let-7b-5p and AURKB relationship has been demonstrated in different cancers. For all these reasons, we concluded that the methods we used would be sufficient to reveal the let-7-5p and AURKB relationship. "P" values were given in the “Statistical analysis section”. After the reviewer's comment, "P" values were also added to the results section.

**o The conclusion posits that the let-7b-5p/AURKB axis is potentially relevant across multiple cancers. However, this claim would be more convincing if supported by data from additional cancer types or a broader range of breast cancer subtypes. Future directions should be clearly outlined, focusing on how these findings could translate into clinical applications, such as miRNA-based therapies.**

**Response:**

We'd like to thank the reviewer for this important feedback. Our investigation revealed the first link between let-7b-5p and AURKB in breast cancer. The let-7b-5p/AURKB relationship has been shown in a few studies in different cancers.

After the reviewer's suggestion, we realized that the relevant sentence was a bit exaggerated. We changed the relevant sentence as follows:

“In conclusion, our findings imply that the let-7b-5p/AURKB/drug resistance link may be in BC. Therefore, it may be vital to conduct deeper investigations on this topic about BC.”

let-7b-5p/AURKB related studies have been discussed in discussion section:

“It has been previously revealed that let-7b-5p directly targets AURKB, reducing the expression of this gene at mRNA and protein levels in different diseases. For instance, Han et al. demonstrated that let-7b-5p reduced cisplatin resistance and tumor development by blocking AURKB in gastric cancer [22]. In another study, Zhou et al. demonstrated that let-7b-5p regulates adriamycin (doxorubicin) resistance via AURKB in chronic myeloid leukemia cells [23]. These findings from the literature demonstrate an important link between let-7b-5p, AURKB, and drug resistance and support our authentic findings between the two molecules.”

Following the reviewer's suggestion, the following section on miRNA-based therapies was added to the discussion section:

“Since miRNAs can target numerous genes, miRNA therapy approach can prove to be more advantageous than the single protein target approach [24]. Due to the ability of these compounds to selectively affect many genes within a complex regulatory network, their activities may result in a more extensive and comprehensive therapeutic response [25]. In vitro and in vivo studies have shown that these molecules possess a substantial potential for the cancer treatment in future [26]. Although miRNA-based therapeutics have potential, they include risks of immunogenicity and safety concerns. The effectiveness of miRNAs specific to targets may be affected by variables like poor RNA stability, uncertain tumor-specific delivery, and localized effects of miRNAs. Despite the advancement of miRNA-targeting medications in clinical trials, none of them have been registered in the clinicaltrials.gov database for the next phase III [26]. It is imperative to have a more precise understanding of the involvement of miRNAs in molecular pathways to solve this challenge. Hence, it is crucial to elucidate the association between miRNAs and their target genes in different types of cancer to facilitate the future utilization of miRNAs as biomarkers and advance the development of therapeutic approaches.”

**Reviewer #2: The study addresses an important clinical challenge in breast cancer treatment: drug resistance, particularly resistance to doxorubicin, a commonly used chemotherapy drug. The exploration of the let-7b-5p/AURKB axis presents a novel target that could enhance the effectiveness of chemotherapy, potentially leading to better patient outcomes; however, there are some points that needs to be considered before publication:**

**1. While the study focuses on the let-7b-5p/AURKB axis, it does not extensively explore other miRNAs or genes that may also play critical roles in doxorubicin resistance, which could limit the comprehensiveness of the findings.**

**Response:**

We'd like to thank the reviewer for this useful feedback. The Bioinformatics approach was used to identify numerous miRNAs that may be related to breast cancer in this study. Indeed, alternative miRNAs and axes including their target genes may also have a role in doxorubicin resistance in breast cancer, and these should be investigated. For example, as noted in the discussion section, let-7c-5p and miR-1290-5p may potentially play a part in this process. Nonetheless, investigating each miRNA/gene axis is a time-consuming and expensive task. Therefore, as the reviewer’s pointed out, the authors of this work concentrated on the let-7b-5p/AURKB axis after the bioinformatics evaluation.

**2. The manuscript suggests potential clinical applications but lacks in-depth discussion on how these findings could be translated into clinical settings or what challenges might be encountered.**

**Response:**

Although miRNAs are regarded as highly fascinating molecules, unfortunately, many challenges have to be solved before miRNAs might be used clinically. Less than twenty miRNA targeting compounds have made it into clinical trials; none advanced to phase III. One of the key issues is, for instance, delivering miRNAs to the cells in the body. Our investigation yielded critical insights about the interaction between miRNA and target genes, which may play a role in breast cancer and doxorubicin resistance. Unfortunately, it seems that these findings cannot be readily translated into therapeutic applications. The following sentences have been added to the discussion section to indicate the difficulties and potentials associated with the use of miRNAs in clinical applications:

“Since miRNAs can target numerous genes, miRNA therapy approach can prove to be more advantageous than the single protein target approach [24]. Due to the ability of these compounds to selectively affect many genes within a complex regulatory network, their activities may result in a more extensive and comprehensive therapeutic response [25]. In vitro and in vivo studies have shown that these molecules possess a substantial potential for the cancer treatment in the future [26]. Although miRNA-based therapeutics have potential, they include risks of immunogenicity and safety concerns. The effectiveness of miRNAs specific to targets may be affected by variables like poor RNA stability, uncertain tumor-specific delivery, and localized effects of miRNAs. Despite the advancement of miRNA-targeting medications in clinical trials, none of them have been registered in the clinicaltrials.gov database for the next phase III [26]. It is imperative to have a more precise understanding of the involvement of miRNAs in molecular pathways to solve this challenge. Hence, it is crucial to elucidate the association between miRNAs and their target genes in different types of cancer to facilitate the future utilization of miRNAs as biomarkers and advance the development of therapeutic approaches.”

**3. The reliance on bioinformatics tools while powerful may introduce biases or limitations inherent to the datasets or tools used such as GEO2R and miRNet. There should be a discussion on potential limitations or biases these tools could introduce.**

**Response:**

We have now discussed this in the manuscript and added the text given below to the discussion section:

“GEO2R is a web-based tool that is user-friendly and enables users to analyze various data sets from a GEO series in order to identify genes, miRNAs, circRNAs, and other molecules that are differentially expressed. Similarly, miRNet [1] is a web-based platform that is user-friendly and has been specifically designed to assist in the elucidation of miRNA effects by integrating users' data with existing knowledge through network-based visual analytics. Nevertheless, it is important to kep in mind that the results obtained using GEO2R, miRNET, and other in silico tools in the study may contain data that could restrict the study. For instance, in the GEO2R analysis, some miRNAs, excluded based on logFC and p-value, may have significant relevance in elucidating the link between BC and doxorubicin resistance. In our work, findings based on miRTarbase and Tarbase tools derived from the miRNET tool used to identify the possible target genes of the chosen miRNA (let-7b-5p). For the prediction of miRNA-Gene interactions, other target genes might be proposed for let-7b-5p in tools other than miRTarbase and Tarbase (e.g. miRDB). Thus, it is important to note that, in addition to the miRNAs and genes identified through various GEO datasets and in silico tools, certain significant miRNAs and genes may have been excluded due to the limitations of the bioinformatics methods employed.”

**4. The study does not include in vivo validation which is crucial to understand how the findings would translate to a whole organism particularly in a clinical context.**

**Response:**

We would like to thank the reviewer for the important criticism. We agree that if our findings could be validated using "in vivo models or patient-derived xenografts," that would be surely excellent. Unfortunately, the project funding does not allow us to integrate these methods within the study. Following the reviewer's recommendation, this limitation has been included in the part on the discussion.

“The investigation provided significant data regarding the relationship between BC Dox resistance/let-7b-5p/AURKB; however, the lack of in vivo experiments represents a different limitation of the study.”

**5. The manuscript would benefit from a more detailed exploration of the functional implications of let-7b-5p/AURKB axis beyond doxorubicin resistance possibly examining other chemotherapeutic agents or combination therapies.**

**Response:**

In the literature, the relationship between Let-7b-5p/AURKB axis and drug resistance has been shown in some cancers. For example, Han et al revealed that the let-7b-5p/AURKB axis is associated with cisplatin resistance in gastric cancer [27]. In a different study, let-7b-5p has been shown to modulate adriamycin resistance in chronic myeloid leukemia K562/ADM cells via AURKB [23].

Nevertheless, the relationship between doxorubicin, AURKB, and let-7b-5p in breast cancer remains unknown. As recommended by the reviewer, new studies can be conducted to investigate the relationship between let-7b-5p/AURKB and other chemotherapeutic agents and/or combination therapies. We would like to express our appreciation and acknowledgement that this significant recommendation from the reviewer will serve as guidance for our future studies.

**6. The discussion on the STRING analysis and the role of overlapping genes could be expanded to provide a deeper understanding of how these genes interact and contribute to cancer progression.**

**Response:**

We would like to thank the reviewer for this valuable contribution. In the Discussion section, genes found to be significant in STRING analysis have been expanded by taking into account situations such as AURKB interaction/BC/drug resistance.

“PPI interaction analysis with STRING revealed that 13 genes have a strong association with AURKB. These genes have been shown to possess potential oncogenic roles in several malignancies, including breast cancer. For instance, Huang et al. showed that CKS2 overexpression is a poor prognostic marker and promotes cell proliferation and invasion in BC [2]. Overexpression of CKS2 has been shown to enhance the effectiveness of chemotherapy by overriding DNA damage regulators [3]. It has been determined that upregulation of NUSAP1 expression stimulates cell proliferation in invasive BC cells via CDK1 and DLGAP5 and is related to Epirubicin resistance [4]. CCNA2 serves as a possible indicator for immunotherapy in BC and acts as a prognostic indicator for ER+ BC and tamoxifen resistance [5, 6]. RRM2 is a prognostic indicator in BC and is associated with worse survival outcomes and tamoxifen resistance [7]. Silencing of RRM2 with siRNA has been shown to significantly inhibit pancreatic tumor proliferation, either alone or with doxorubicin [8]. CDCA8 is another prominent gene that may be related to AURKB. It is a key modulator of estrogen-induced cell proliferation in BC cells [9]. The AURKB/CDCA8 pathway is potentially pivotal in cancer pathogenesis. Hayama et al. showed that the phosphorylation and activation of CDCA8 by AURKB contribute to human lung carcinogenesis [10]. PLK1, whose increased expression is a major problem in BC, has been reported to mediate resistance to Palbociclib in HR+/HER2- metastatic BC [11]. According to studies, BIRC5 (surviving), an inhibitor of apoptosis (IAP) family member was substantially expressed in BC patients and, it also promotes resistance to chemotherapy, anti-HER2 treatment, and radiation [12]. Mitotic kinases PLK1 and AURKB control BC cell proliferation by phosphorylating BIRC5 [13]. The literature compiled here indicate that by clarifying the molecular processes of genes that interact strongly with AURKB in STRING analysis, drug resistance mechanisms in breast cancer can be better understood and new treatment strategies can be developed.”

**7. Some sections particularly the introduction and discussion could benefit from more scientific language and a stronger focus on the most critical findings. Redundant detailed explanations of background information should be minimized.**

**Response:**

We thank the reviewer for this valuable comments. Required updates have been made to the introduction and discussion sections.

**8. There is a need for a more explicit linkage between the study’s findings and the potential clinical applications providing a clearer pathway for future research.**

**Response:**

Although miRNAs are seen to be extremely promising molecules, several challenges must be addressed before they can be employed in clinical applications. The difficulties in identifying miRNA targets, the poor potency of miRNA inhibitors, cell type-specific delivery, and some adverse outcomes have slowed the development of miRNA therapies. These challenges arise from the functional complexities of miRNA's participation in illness and sequence complementarity-dependent/independent actions in nontarget tissues. However, it is thought that more detailed elucidation of the miRNA-disease relationship, a clearer understanding of the relationship between miRNA target genes, the development of effective miRNA inhibitors and innovative application techniques may help overcome some of these difficulties.

**9. The language of overall manuscript needs to be more scientific and grammatical errors needs to be checked thoroughly.**

**Response:**

We express our gratitude to the reviewer for this insightful comment. Following the reviewer's recommendation, the manuscript was re-evaluated and the appropriate revisions were carried out.

**10. All the images should be revised in higher resolution of 600 dpi as the quality is very poor.**

**Response:**

The image quality was reduced in the PDF built by the journal’s editorial system.

We would like to kindly remind the reviewers that the editorial system generates a deteriorated, low-quality version of the images while merging all uploaded files but, on the top right corner of each image there is a link to download the original, high quality, image. E.g. for Figure 11:

**Reviewer #3: Here, in this work authors identified target for miRNA let-7b-5p, and used AURKB as a target to interpret the drug resistance. Some comments related to are**

**1. Include a figure to represent the steps used in the identification of differentially expressed MiRNA.**

**Response:**

Thanks for the useful comment. Figure 11 shows the steps of the detected differentially expressed MiRNAs and Genes. It was probably missed due to the low quality of the image we provided. Upon reviewer's suggestion, Figure 11 was modified and the image quality was increased to 1200 dpi. We would like to kindly remind the reviewers that the editorial system generates a deteriorated, low-quality version of the images while merging all uploaded files but, on the top right corner of each image there is a link to download the original, high quality, image. For example for Figure 11:

**2. What actually logFC calculates?**

**Response:**

We'd like to thank you to the reviewer for their helpful comment. The logFC explanation has now been added to the material & method section, as narrated below.

“The differential expression has been defined as an alteration in the log-fold change (logFC). A log-fold change (logFC) >1.0 indicates that the miRNAs or genes have changed at least twice (fold change >2.0), suggesting significant differences across groups. In this study miRNAs and Genes were considered differentially expressed if |logFC| > 1 or |logFC| > 2 respectively.”

**3. Check italics case and abbreviation.**

**Response:**

Required corrections have been made to the manuscript. Thank you.

**4. Discuss the other genes that are in connection/interaction with AURKB gene.**

**Response:**

Thank you to the reviewer for their insightful feedback. Other genes that are in connection/interaction with AURKB gene were discussed in the discussion section:

“In current study a comparison of the possible target genes of let-7b-5p in the miRNet tool between TCGA-BC data, GSE100925, and GSE229571 BC GEO datasets revealed 14 genes in common. The STRING analysis revealed a strong link between these 14 genes. Furthermore, a significant association was identified between AURKB and the other 13 genes. These genes have been shown to possess potential oncogenic roles in several malignancies, including BC. For instance, Huang et al. showed that CKS2 overexpression is a poor prognostic marker and promotes cell proliferation and invasion in BC [2]. Overexpression of CKS2 has been shown to enhance the effectiveness of chemotherapy by overriding DNA damage regulators [3]. It has been determined that upregulation of NUSAP1 expression stimulates cell proliferation in invasive BC cells via CDK1 and DLGAP5 and is related to Epirubicin resistance [4]. CCNA2 serves as a possible indicator for immunotherapy in BC and acts as a prognostic indicator for ER+ BC and tamoxifen resistance [5, 6]. RRM2 is another prognostic indicator in BC and is associated with worse survival outcomes and tamoxifen resistance [7]. RRM2 silencing with siRNA has been shown to significantly inhibit pancreatic tumor proliferation, either alone or with Dox [8]. CDCA8 is another prominent gene that may be related to AURKB. It is a key modulator of estrogen-induced cell proliferation in BC cells [9]. The AURKB/CDCA8 pathway is potentially pivotal in cancer pathogenesis. Hayama et al. showed that the phosphorylation and activation of CDCA8 by AURKB contribute to human lung carcinogenesis [10]. PLK1, whose increased expression is a major problem in BC, has been reported to mediate resistance to Palbociclib in HR+/HER2- metastatic BC [11]. According to studies, BIRC5 (surviving), an inhibitor of apoptosis (IAP) family member was substantially expressed in BC patients and, it also promotes resistance to chemotherapy, anti-HER2 treatment, and radiotherapy [12]. Mitotic kinases PLK1 and AURKB control BC cell proliferation by phosphorylating BIRC5 [13]. The literature that was assembled here suggests that the molecular linkage of genes connected to AURKB should be examined in more depth. This would allow for a better understanding of the processes that cause drug resistance in BC, as well as the development of novel treatments. Based on these findings, it has been suggested that the let-7b-5p/AURKB axis may affect cancer processes by influencing several key genes.”

**5. Quality of figure 11 is very poor and blurred. Please provide a good quality figures.**

**Response:**

After reviewer comments, figure 11 had been modified and also, the image quality was increased to 1200 dpi. However we would like to kindly remind the reviewers that the editorial system generates a deteriorated, low-quality version of the images while merging all uploaded files but, on the top right corner of each image there is a link to download the original, high quality, image. For example:

**6. Is AURAKB target for Breast cancer therapy only ? or for other types cancers also.**

**Response:**

AURKB is a gene that is overexpressed in many cancers. Therefore, one can speculate that an AURKB-related treatment strategy may be successful not only in breast cancer but also in different cancers. Some discussions on the relevant studies added to the article upon the relationship between AURKB and cancers are as follows:

“The AURKB gene is overexpressed in many cancer types including BC [14-16]. Pellizzari et al reported that inhibition of the AURKB pathway may be a promising strategy for the treatment and radiosensitization of Triple Negative Breast Cancer (TNBC) [17]. It has been demonstrated that AURKB stimulates epithelial-mesenchymal transition by stabilizing Snail1, resulting in basal-like breast cancer metastases [18]. AURKB is also closely associated with drug resistance. For instance, Liu et al., revealed that AURKB is related to paclitaxel resistance in BC cells through the PRKCE/RAB27B axis, suggesting a possible intervention target to reverse tumor drug resistance [19]. A different investigation has demonstrated that AURKB facilitates the development of tumors and carboplatin resistance via controlling the ERK pathway in neuroblastoma cells [20]. Some studies have also shown that miRNAs can specifically target AURKB and are associated with drug resistance in cancers. For example, miR-486-5p has been shown to regulate DNA damage inhibition and cisplatin resistance in lung adenocarcinoma via AURKB [21]. The results of these studies indicate that AURKB may be responsible for drug resistance in certain malignancies through various molecular mechanisms, such as miRNAs. The provided research supports that let-7b-5p may be linked to doxorubicin resistance in BC through AURKB.”

1.	Chang, L., G. Zhou, O. Soufan, and J. Xia, *miRNet 2.0: network-based visual analytics for miRNA functional analysis and systems biology.* Nucleic Acids Res, 2020. **48**(W1): p. W244-w251.

2.	Huang, N., et al., *Overexpression of CKS2 is associated with a poor prognosis and promotes cell proliferation and invasion in breast cancer.* Mol Med Rep, 2019. **19**(6): p. 4761-4769.

3.	del Rincón, S.V., et al., *Cks overexpression enhances chemotherapeutic efficacy by overriding DNA damage checkpoints.* Oncogene, 2015. **34**(15): p. 1961-7.

4.	Zhang, X., Y. Pan, H. Fu, and J. Zhang, *Nucleolar and Spindle Associated Protein 1 (NUSAP1) Inhibits Cell Proliferation and Enhances Susceptibility to Epirubicin In Invasive Breast Cancer Cells by Regulating Cyclin D Kinase (CDK1) and DLGAP5 Expression.* Med Sci Monit, 2018. **24**: p. 8553-8564.

5.	Wang, Y., et al., *Integrated Profiling Identifies CCNA2 as a Potential Biomarker of Immunotherapy in Breast Cancer.* Onco Targets Ther, 2021. **14**: p. 2433-2448.

6.	Gao, T., et al., *CCNA2 is a prognostic biomarker for ER+ breast cancer and tamoxifen resistance.* PLoS One, 2014. **9**(3): p. e91771.

7.	Putluri, N., et al., *Pathway-centric integrative analysis identifies RRM2 as a prognostic marker in breast cancer associated with poor survival and tamoxifen resistance.* Neoplasia, 2014. **16**(5): p. 390-402.

8.	Zheng, S., et al., *siRNA Knockdown of RRM2 Effectively Suppressed Pancreatic Tumor Growth Alone or Synergistically with Doxorubicin.* Mol Ther Nucleic Acids, 2018. **12**: p. 805-816.

9.	Bu, Y., et al., *CDCA8 is a key mediator of estrogen-stimulated cell proliferation in breast cancer cells.* Gene, 2019. **703**: p. 1-6.

10.	Hayama, S., et al., *Phosphorylation and activation of cell division cycle associated 8 by aurora kinase B plays a significant role in human lung carcinogenesis.* Cancer Res, 2007. **67**(9): p. 4113-22.

11.	Guerrero-Zotano, Á., et al., *CCNE1 and PLK1 Mediate Resistance to Palbociclib in HR+/HER2- Metastatic Breast Cancer.* Clin Cancer Res, 2023. **29**(8): p. 1557-1568.

12.	Adinew, G.M., S. Messeha, E. Taka, and K.F.A. Soliman, *The Prognostic and Therapeutic Implications of the Chemoresistance Gene BIRC5 in Triple-Negative Breast Cancer.* Cancers (Basel), 2022. **14**(21).

13.	Garlapati, C., et al., *PLK1 and AURKB phosphorylate survivin differentially to affect proliferation in racially distinct triple-negative breast cancer.* Cell Death Dis, 2023. **14**(1): p. 12.

14.	Li, L., et al., *AURKB promotes bladder cancer progression by deregulating the p53 DNA damage response pathway via MAD2L2.* J Transl Med, 2024. **22**(1): p. 295.

15.	Wang, H., H. Pan, and X. Huang, *AURKB Enhances Chromosomal Remodeling of Telomeric Genes and Accelerates Tumorigenesis of Uveal Melanoma.* Invest Ophthalmol Vis Sci, 2023. **64**(4): p. 23.

16.	Kaya, M., et al., *Overexpression of CDC25A, AURKB, and TOP2A Genes Could Be an Important Clue for Luminal A Breast Cancer.* Eur J Breast Health, 2024. **20**(4): p. 284-291.

17.	Pellizzari, S., H. Athwal, A.C. Bonvissuto, and A. Parsyan, *Role of AURKB Inhibition in Reducing Proliferation and Enhancing Effects of Radiotherapy in Triple-Negative Breast Cancer.* Breast Cancer (Dove Med Press), 2024. **16**: p. 341-346.

18.	Zhang, J., et al., *Aurora B induces epithelial-mesenchymal transition by stabilizing Snail1 to promote basal-like breast cancer metastasis.* Oncogene, 2020. **39**(12): p. 2550-2567.

19.	Liu, M., Y. Li, C. Zhang, and Q. Zhang, *Role of aurora kinase B in regulating resistance to paclitaxel in breast cancer cells.* Hum Cell, 2022. **35**(2): p. 678-693.

20.	Yang, Y., et al., *AURKB promotes tumorigenesis and carboplatin resistance by regulating the ERK pathway in neuroblastoma cells.* Int J Neurosci, 2023. **133**(11): p. 1224-1232.

21.	Sun, G., et al., *microRNA-486-5p Regulates DNA Damage Inhibition and Cisplatin Resistance in Lung Adenocarcinoma by Targeting AURKB.* Crit Rev Eukaryot Gene Expr, 2024. **34**(4): p. 13-23.

22.	Han, X., et al., *Let-7b attenuates cisplatin resistance and tumor growth in gastric cancer by targeting AURKB.* Cancer Gene Ther, 2018. **25**(11-12): p. 300-308.

23.	Zhou, X., et al., *Let-7b regulates the adriamycin resistance of chronic myelogenous leukemia by targeting AURKB in K562/ADM cells.* Leuk Lymphoma, 2020. **61**(14): p. 3451-3459.

24.	Kaya, M., *Bioinformatics evaluation of the circRNA–miRNA–mRNA axis in cervical squamous cell carcinoma.* Exploration of Medicine, 2024. **5**: p. 553-565.

25.	Mishra, S., T. Yadav, and V. Rani, *Exploring miRNA based approaches in cancer diagnostics and therapeutics.* Crit Rev Oncol Hematol, 2016. **98**: p. 12-23.

26.	Pagoni, M., et al., *miRNA-Based Technologies in Cancer Therapy.* J Pers Med, 2023. **13**(11).

27.	Han, X., H.B. Zhang, X.D. Li, and Z.A. Wang, *Long non-coding RNA X-inactive-specific transcript contributes to cisplatin resistance in gastric cancer by sponging miR-let-7b.* Anticancer Drugs, 2020. **31**(10): p. 1018-1025.
